# Spatial genome organization: contrasting views from chromosome conformation capture and fluorescence in situ hybridization

**DOI:** 10.1101/gad.251694.114

**Published:** 2014-12-15

**Authors:** Iain Williamson, Soizik Berlivet, Ragnhild Eskeland, Shelagh Boyle, Robert S. Illingworth, Denis Paquette, Josée Dostie, Wendy A. Bickmore

**Affiliations:** 1 MRC Human Genetics Unit, Institute of Genetics and Molecular Medicine, University of Edinburgh, Edinburgh EH4 2XU, United Kingdom;; 2 Department of Biochemistry,; 3 Goodman Cancer Research Center, McGill University, Montréal, Québec H3G1Y6, Canada

**Keywords:** 3C, FISH, Hox genes, nuclear organization, polycomb

## Abstract

Although important for gene regulation, most studies of genome organization use either fluorescence in situ hybridization (FISH) or chromosome conformation capture (3C) methods. FISH directly visualizes the spatial relationship of sequences but is usually applied to a few loci at a time. The frequency at which sequences are ligated together by formaldehyde cross-linking can be measured genome-wide by 3C methods, with higher frequencies thought to reflect shorter distances. FISH and 3C should therefore give the same views of genome organization, but this has not been tested extensively. We investigated the murine *HoxD* locus with 3C carbon copy (5C) and FISH in different developmental and activity states and in the presence or absence of epigenetic regulators. We identified situations in which the two data sets are concordant but found other conditions under which chromatin topographies extrapolated from 5C or FISH data are not compatible. We suggest that products captured by 3C do not always reflect spatial proximity, with ligation occurring between sequences located hundreds of nanometers apart, influenced by nuclear environment and chromatin composition. We conclude that results obtained at high resolution with either 3C methods or FISH alone must be interpreted with caution and that views about genome organization should be validated by independent methods.

## Abstract (precis)

Williamson et al. investigate the murine *HoxD* locus with 5C and FISH in different developmental and activity states and in the presence or absence of epigenetic regulators. They identify situations in which the two data sets are concordant but find other conditions under which chromatin topographies extrapolated from 5C or FISH data are not compatible. Products captured by 3C do not always reflect spatial proximity, with ligation occurring between sequences located hundreds of nanometers apart, influenced by nuclear environment and chromatin composition.

Fluorescence microscopy has been instrumental to our understanding of the spatial organization of the genome. Detection of chromosomes by fluorescence in situ hybridization (FISH) in either fixed cells (Cremer and Cremer 2006) or living cells (Edelmann et al. 2001; Müller et al. 2010) reveals specific chromosome territories (CTs) that are nonrandomly organized in vertebrate nuclei. Gene-rich chromosomes generally cluster at the nuclear center, while gene-poor chromosomes and chromatin domains often locate near the nuclear periphery (Croft et al. 1999; Boyle et al. 2001, 2011; Küpper et al. 2007; Kölbl et al. 2012). This level of genome organization is stochastic: Loci at the nuclear periphery in a mother cell can localize to either the nuclear or nucleolar periphery in daughter nuclei (Thomson et al. 2004; Kind et al. 2013).

Cytologically, gene-dense chromatin containing active loci is observed to decondense and dynamically loop out from CT cores both at individual loci (Mahy et al. 2002; Chambeyron and Bickmore 2004; Müller et al. 2010) and chromosome-wide (Boyle et al. 2011). A propensity for clustering of gene-rich chromatin domains from the same or different chromosomes is also seen (Shopland et al. 2006; Brown et al. 2008).

FISH also identifies differences in chromatin condensation at the submegabase level, between genomic regions in the same cell (Yokota et al. 1997; Gilbert et al. 2004), for a given region during differentiation in vitro (Chambeyron and Bickmore 2004; Morey et al. 2007) and in vivo (Williamson et al. 2012; Patel et al. 2013), or between wild-type and mutant cells (Eskeland et al. 2010; Nolen et al. 2013). FISH has also been used to examine tissue-specific colocalization of long-range enhancers and their target genes (Amano et al. 2009; Williamson et al. 2012)

Although visually compelling, FISH and live-cell imaging are restricted to viewpoints corresponding to the regions detected by the probes used (Dostie and Bickmore 2012), are low-throughput assays, and have limited spatial resolution, although superresolution microscopy is improving the latter (Markaki et al. 2012; Nora et al. 2012; Patel et al. 2013). In contrast, the chromosome conformation capture (3C) technique and its derivatives—including circular 3C (4C), 3C carbon copy (5C), and chromosome capture followed by high-throughput sequencing (Hi-C) (for review, see de Wit and de Laat 2012; Ethier et al. 2012)—offer a genome-wide perspective on genome organization. By digesting and ligating chromatin within formaldehyde cross-linked cells, these methods measure the frequency at which sequences are joined together by ligation. Unlike imaging in which distances are measured directly in individual cells, 3C methods infer physical proximity by considering that ligation frequency is more or less inversely proportional to the original spatial separation of the two sequences (Fraser et al. 2009; Ferraiuolo et al. 2010; Baù and Marti-Renom 2011; Noordermeer et al. 2011; Wang et al. 2011).

The limited resolution of early Hi-C studies (∼1 Mb) (Lieberman-Aiden et al. 2009; Kalhor et al. 2011) gave coarse-grained views of nuclear organization that agree well with microscopy data, reproducing the existence of CTs, the spatial clustering of gene-rich chromosomes in the nuclear center, looping out of gene-rich domains from CTs, and the tendency for active genomic domains to associate. Whereas FISH is inherently a single-cell/allele technique, most Hi-C analyses give data sets of ligation frequencies averaged across all cells in a population. A single-cell Hi-C study recently overcame this limitation, albeit at the expense of resolution (Nagano et al. 2013), and confirmed the stochastic nature of CT architecture and the general disposition of active chromatin to reside toward the outside of CTs.

More recently, higher-resolution Hi-C and 5C studies have suggested that mammalian genomes are organized into globules (Baù and Marti-Renom 2011) or domains (topologically associating domains [TADs]) within which cross-linked associations are enriched (Dixon et al. 2012; Nora et al. 2012; Jin et al. 2013; Giorgetti et al. 2014). The largely invariant nature of TAD boundaries between different cell types suggests that they reflect a basic property of genome organization. Indeed, the average size of these domains (∼900 kb) is similar to the ∼1-Mb domains suggested from microscopy to form the basic building blocks (∼500-nm diameter) of CT architecture (Albiez et al. 2006; Baù and Marti-Renom 2011). FISH showed that sequences within a TAD intermingle to a much greater extent than those that span adjacent TADs (Nora et al. 2012).

In addition to providing insight into global chromatin organization, 3C methods have been used to identify associations between specific sequences involved in gene regulation—including silent *Hox* gene clusters regulated by the polycomb-repressive complexes (PRCs) (Fraser et al. 2009; Ferraiuolo et al. 2010; Noordermeer et al. 2011; Wang et al. 2011), domains of clustered active genes (*Hox* and *α-globin* clusters) (Baù et al. 2011; Noordermeer et al. 2011; Wang et al. 2011), and “looping” between long-range enhancers and their target genes (Montavon et al. 2011; Sanyal et al. 2012; Andrey et al. 2013; Berlivet et al. 2013; Hughes et al. 2014)—and between sites bound by proteins involved in chromosome architecture (Philips-Cremins et al. 2013; Seitan et al. 2013; Sofueva et al. 2013; Zuin et al. 2014).

By supposing that high-frequency contacts are sequences close to each other in vivo, data from 3C experiments have been used to reconstruct the likely trajectory of chromatin fibers and relate chromatin folding to gene regulation (Fraser et al. 2009; Baù and Marti-Renom 2011; Montavon et al. 2011; Giorgetti et al. 2014). While the resulting models are informative, some are not consistent with the views assayed by FISH (Dostie and Bickmore 2012; Williamson et al. 2012). This points to differences in what 3C methods and FISH can detect and demands a careful comparison of molecular and cytological data from the same experimental system. In most studies that have attempted to do this, interprobe FISH distances up 500 nm (sometimes even more) have been taken as evidence validating 3C interactions. However, such comparatively large physical separations do not seem compatible with interactions at the molecular scale (Belmont 2014). Here we investigate chromatin organization of the murine *HoxD* locus with 5C and three-dimensional (3D) FISH in different developmental and activity states and after perturbing epigenetic regulation. We identified some conditions under which the standard interpretation of 5C data as a measure of spatial proximity produces views of chromatin topography that are not compatible with those obtained by FISH. We conclude that results obtained by either high-resolution 3C-type experiments or FISH alone must be interpreted with caution when studying chromatin architecture.

## Results

### Topography of HoxD in embryonic stem cells (ESCs)

In terms of chromatin organization, the *Hox* gene clusters are among the most extensively studied regions in mammalian cells. By FISH, they have a visibly compact conformation in mouse ESCs (mESCs), where they are maintained in an inactive state by the PRCs (Chambeyron and Bickmore 2004; Morey et al. 2007; Eskeland et al. 2010). This is also seen in mouse embryonic development (Chambeyron et al. 2005; Morey et al. 2007; Ku et al. 2008; Montavon et al. 2011). These observations are compatible with the condensed appearance on polytene chromosomes of the bithorax complex, one of the two *Hox* clusters in *Drosophila* (Marchetti et al. 2003), mitigating concerns about artifacts due to the FISH procedure. A relatively compact chromatin domain with multiple interactions within it is also predicted from 3C analysis of silent *HOX* loci in human embryonal carcinoma (EC) cells (Simonis et al. 2006; Ferraiuolo et al. 2010) and 4C analysis of silent *Hox* loci in mESCs and mouse embryos (Montavon et al. 2011; Noordermeer et al. 2011, 2014).

**Figure 1.**
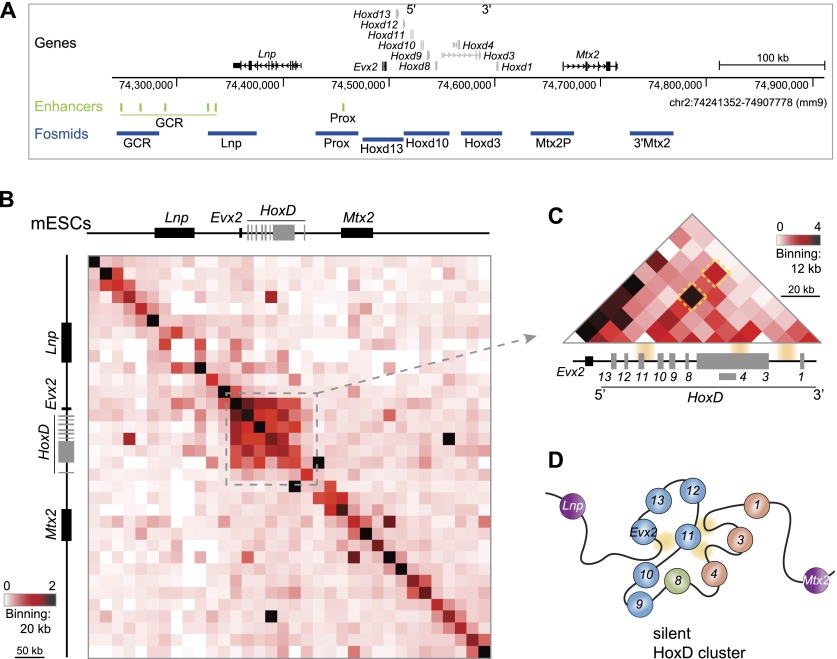
5C analysis of *HoxD* in ESCs. (*A*) A 670-kb region analyzed by 5C in OS25 mESCs, encompassing *Mtx2*, *Hoxd* genes, *Evx2*, and *Lnp*. Distal regulatory elements (Montavon et al. 2011) are highlighted in green. Positions of FISH probes are indicated in blue. Genome coordinates are from the NCBI37/mm9 assembly of the mouse genome. (*B*) Analysis of chromatin organization in undifferentiated mESCs by 5C sequencing across the 670-kb region shown in *A*. The heat map shows 5C data binned over 20-kb windows. Heat map intensities represent the average of interaction frequency for each window, color-coded according to the scale shown. Data for a biological replicate are in Supplemental Figure S1A. Unprocessed normalized data are shown in Supplemental Figure S7. All interaction frequencies were first normalized based on the total number of sequence reads in the 5C data set. (*C*) High-resolution (12-kb binning) zoomed-in view of the 5C data over *Exv2* and the *HoxD* locus. The two contacts conserved in human embryonic carcinoma cells are indicated with dashed yellow boxes. (*D*) Two-dimensional schematic interpretation of the 5C data in OS25 mESCs illustrating the folded nature of the *Evx2–HoxD* domain (not to scale). Contacts between *Hoxd11* and regions downstream from *d1*, *d3*, and *Evx2* are highlighted in yellow.

In contrast, the lack of long-range interactions captured by 5C at silent parts of *HOXA* in human fibroblasts was suggested to reflect an extended linear chromatin domain (Wang et al. 2011). Barring any source of technical bias, this result either reflects tissue-specific organization of silent *HOX* genes or might point to differences between what is sometimes captured with 3C methods and by FISH. We first investigated whether FISH and 5C invariably yield similar views of chromatin organization by examining a ∼670-kb region containing the inactive *HoxD* cluster (Fig. 1A) in undifferentiated mESCs. A prominent feature is the high level of 5C interactions detected within the *HoxD*–*Evx2* region (Fig. 1B; Supplemental Figs. S1A, S6A**)**. The pattern of these 5C contacts (Fig. 1C; Supplemental Fig. S1B) is similar to the one that we reported for the silent *HOXD* cluster in human EC cells (Ferraiuolo et al. 2010), supporting a conserved chromatin conformation at *HoxD* in pluripotent stem cells. If interaction frequency is viewed as inversely proportional to spatial distance, our 5C analysis indicates that the region encompassing the *Hoxd* and *Evx2* genes is folded into a structure that is the most compact part of the region under study (Fig. 1D). This is consistent with our previous observation that the silent *HoxD* cluster in mESCs adopts a visibly compact structure by FISH (Morey et al. 2007).

**Figure 2.**
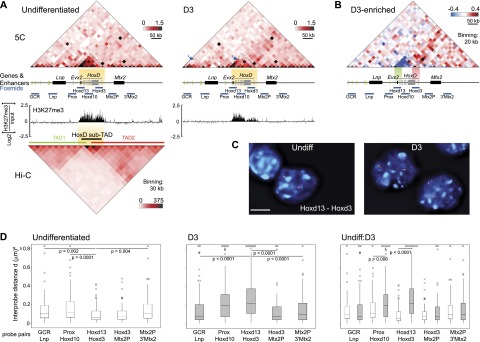
Decompaction of the *HoxD* cluster and increased long-range interactions accompany ESC differentiation. (*A*) Analysis of chromatin organization during OS25 mESC differentiation by 5C sequencing across a 670-kb region containing *Lnp*, *Evx2*, *HoxD* genes, and *Mtx2* in undifferentiated ESCs (*left*) and differentiated cells (D3; *right*). Blue arrows point to GCR–*Lnp* contacts and *HoxD* contacts to compare interaction frequencies. The *top* heat maps show 5C data binned over 20-kb windows, and heat map intensities represent the average interaction frequency for each window. Interaction frequencies were normalized based on total sequence read number. Yellow shading indicates the region of strong 5C signals. H3K27me3 ChIP–chip data (Eskeland et al. 2010) are presented *below*. The *bottom left* heat map shows Hi-C data at 30-kb resolution, normalized based on read depth (Dixon et al. 2012), and TADs for the corresponding region in mESCs. Data for a biological replicate are in Supplemental Figure S1. Unprocessed normalized data are shown in Supplemental Figure S6A. (*B*) Heat map showing interactions enriched in D3 differentiated (red) or undifferentiated (blue) ESCs. Heat map values represent the difference of normalized interaction frequencies between D3 differentiated and undifferentiated cells as indicated by the color scale at the *right*. Green and red shading highlight the regions covered by the fosmids used for the analysis in *C*. (*C*) 3D-FISH with Hoxd13 and Hoxd3 probe pairs counterstained with DAPI (blue) in nuclei from undifferentiated (Undiff) and differentiated (D3) ESCs. Bars, 5 μm. (*D*) Box plots showing the distribution of squared interprobe distances (*d*^2^) in micrometers for GCR–Lnp, Prox–Hoxd10, Hoxd13–Hoxd3, Hoxd3–Mtx2P, and Mtx2P–3′Mtx2 FISH probe pairs in undifferentiated (*left*) and D3 (*middle*) mESCs. The *right* box plots compare the interprobe distance distributions for each probe pair in undifferentiated (Undiff) versus D3 ESCs. Boxes show the median and interquartile range of the data; crosses signify outliers. *n* = 86–101 loci. The statistical significance between data sets was examined by Mann-Whitney *U*-tests. These data are plotted as histograms of the frequency distribution in Supplemental Figure S2.

The extent of the strong 5C long-range contacts across *HoxD* in mESCs corresponds well to the domain of histone H3 Lys27 trimethylation (H3K27me3) deposited by the PRC2 complex (Eskeland et al. 2010) and to the sub-TAD identified at *Evx2*–*HoxD* by Hi-C in mESCs (Fig. 2A; Dixon et al. 2012). At our sequencing depth, we did not see evidence of the two TADs that extend 3′ and 5′ of *HoxD* in the published Hi-C analysis and saw little evidence of other significant long-range 5C interactions in the regions flanking *HoxD*.

### Reorganization of HoxD upon ESC differentiation

Retinoic acid (RA)-directed differentiation of mESCs results in activation of *Hoxd* genes (Morey et al. 2007) and visible chromatin decompaction of the locus (Eskeland et al. 2010) reminiscent of the puffed appearance of the active bithorax locus in *Drosophila* polytene chromosomes (Marchetti et al. 2003). However, this contrasts with views of activated *Hox* loci extrapolated from 5C analysis of human fibroblasts (Wang et al. 2011) and 4C in mouse embryos (Noordermeer et al. 2011, 2014). From those data, it was suggested that active *Hox* genes form compact domains.

To determine whether these contrasting models stem from inherent differences in what FISH and 3C techniques can detect, we examined chromatin organization across *HoxD* with 5C and FISH in mESCs differentiated for 3 d (D3) with RA (Fig. 2A; Supplemental Figs. S1A, S6A). Under these conditions, gene activation and loss of H3K27me3 mainly occur in the 3′ part of *HoxD* (Fig. 2A; Morey et al. 2007; Eskeland et al. 2010). The high 5C interactions across *HoxD* that we detected in undifferentiated mESCs are strongly reduced in D3 cells but, like H3K27me3, are not completely lost (Fig. 2A,B; Supplemental Fig. S1C). This is consistent with the visible unfolding of *HoxD* chromatin seen upon mESC differentiation (Fig. 2C,D) and the loss of long-range 3C and 5C contacts reported upon *HOXA* activation (Fraser et al. 2009; Ferraiuolo et al. 2010).

FISH using probe pairs across the whole region interrogated by 5C (Supplemental Table S1) confirmed a visible chromatin decompaction during differentiation and that this is restricted to the region overlapping *HoxD* and *Evx2* (*Hoxd13*–*Hoxd3; Prox*–*Hoxd10*) (Fig. 2D; Supplemental Tables S2, S3). However, although FISH indicates that the *HoxD* cluster itself is by far the most unfolded part of this entire 700-kb region in D3 cells, the capture frequencies of many 5C fragment pairs within *HoxD* are still comparable with, or greater than, those in the flanking regions. For example, the global control region (GCR)–*Lnp* region 5′ of *HoxD* is more visibly compact than *HoxD* in D3 cells (Fig. 2D) but has lower 5C reads (Fig. 2A, arrow; Supplemental Fig. S1A).

Apart from the loss of interactions within *HoxD*, the other major change in the 5C profile is the increased interactions captured during differentiation between 3′ *Hoxd* genes and the region extending through *Mtx2* and into the gene desert beyond (Fig. 2B; Supplemental Fig. S1C). However, the distribution of FISH interprobe distances across these regions (*Hoxd3*–*Mtx2P* and *Mtx2P*–*3′Mtx2*) remains largely unchanged upon differentiation (Fig. 2D; Supplemental Fig. S2; Supplemental Tables S2–4), suggesting no major change in chromatin compaction.

These data caution against the simple assumption that spatial proximity between genomic regions can always be inferred at high resolution from either contact frequencies in 3C-based assays or FISH alone.

### Topography of the HoxD regulatory domain across the anterior–posterior axis of the distal limb

**Figure 3.**
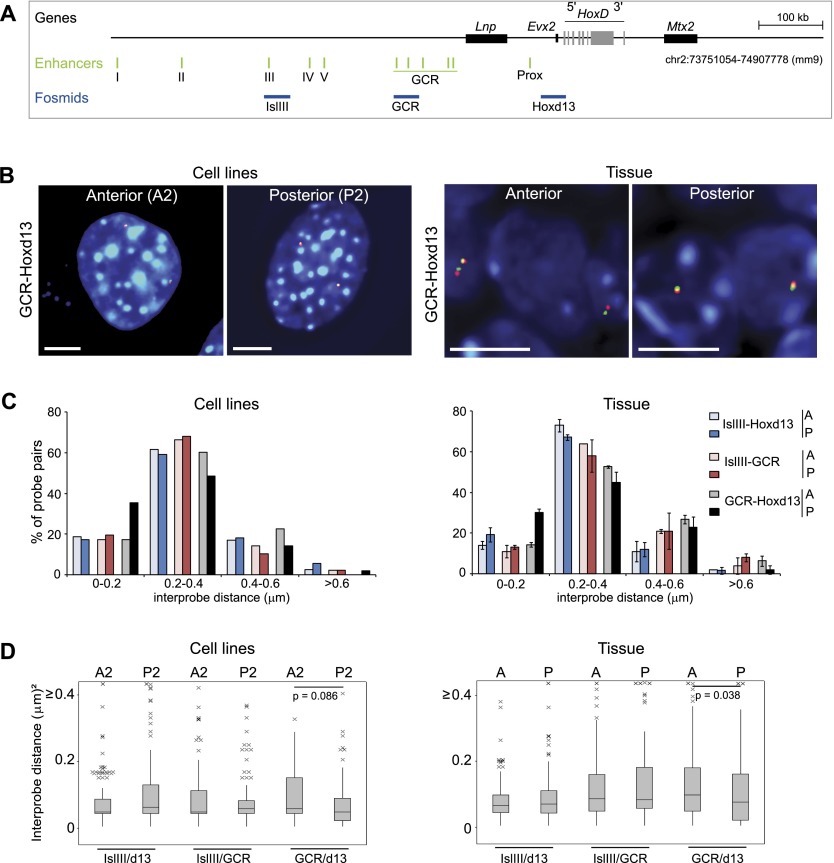
FISH analysis of the 5′ *HoxD* regulatory region in anterior and posterior distal limb cells. (*A*) A 1.16-Mb region analyzed by 5C in limb cells and including the gene desert 5′ of *HoxD* that contains distal limb-specific regulatory elements (highlighted in green) (Montavon et al. 2011). Positions of the fosmids used for FISH experiments are indicated in blue. (*B*) FISH with Hoxd13 and GCR probes in nuclei of cell lines derived from the anterior (A2) or posterior (P2) E10.5 distal embryonic forelimb (*left*) and anterior or posterior E11.0 distal forelimb tissue (*right*). Bars, 5 μm. (*C*) Frequency distributions of interprobe distances (*d*) in 0.2-μm bins between the GCR, Hoxd13, and IslIII probes in nuclei from A2 and P2 limb cell lines (*left*) or anterior (A) and posterior (P) parts of the distal E11.0 forelimb bud (*right*) (data from Williamson et al. 2012). *n* = 93–151. Error bars represent SEM obtained from two different tissue sections. (*D*) Box plots *below* show the distribution of squared interprobe distances (*d*^2^) in micrometers between IslIII/Hoxd13, IslIII/GCR, and GCR/Hoxd13 probe pairs in A2 and P2 limb cell lines (*left*) and anterior (A) or posterior (P) limb tissue (*right*). *n* = 191 loci for cell lines; *n* = ∼100 loci for tissue. The statistical significance of differences between data from distal anterior and distal posterior nuclei was examined by Mann-Whitney *U*-tests.

To gain more insight into views of long-range chromatin conformation from 3C and FISH, we examined *HoxD* in another developmental context. Digit growth and patterning depend on the correct regulation of expression from 5′ *Hoxd* genes, initiated in the posterior part of the distal limb bud during the late phase of limb development. This is controlled by multiple long-range regulatory elements/enhancers spread throughout a large gene desert 5′ of *HoxD* (Fig. 3A; Spitz et al. 2003; Montavon et al. 2011). In 4C assays from embryonic day 12.5 (E12.5) distal limb buds, these enhancers could be captured cross-linked to both the promoter of *Hoxd13* and each other. It was therefore suggested that these elements are brought close to each other and *Hoxd13* by multiple chromatin loops.

Consistent with this model, FISH in the E11 distal forelimb reveals a posterior-specific high (30%) colocalization (≤200 nm) frequency of *Hoxd13* alleles, with the best-characterized regulatory element 180 kb upstream of *Hoxd13*—the GCR (Fig. 3B,C; Supplemental Table S5; Williamson et al. 2012). This is recapitulated in mesenchymal cell lines derived from the anterior (A2) or posterior (P2) of dissected E10.5 forelimb buds (Fig. 3B,C), which also preserve the anterior–posterior differences in 5′ *Hoxd* expression (Supplemental Fig. S3; Williamson et al. 2012). The *Hoxd13*–GCR colocalization frequency (35%) in P2 cells, which strongly express *Hoxd13*, is significantly higher (*P* = 0.005) than that in A2 cells (Fig. 3C; Supplemental Table S5). However, as we reported previously in limb tissue (Williamson et al. 2012), there is little colocalization between *Hoxd13* and another of the regulatory elements, island III (IslIII), or between GCR and IslIII in A2 and P2 cells, and there is no elevated colocalization frequency in P2 cells compared with A2 (Fig. 3C; Supplemental Table S5). This argues against a model in which the different enhancers are brought together by discrete contacts into a regulatory hub (Montavon et al. 2011). Rather, the low median *Hoxd13*–IslIII and GCR–IslIII interprobe FISH distances (221–250 nm) (Fig. 3D; Supplemental Table S6) suggest that the whole region from IslIII to *Hoxd13* is configured into a generally compact structure in both cell lines and tissue from the distal limb.

**Figure 4.**
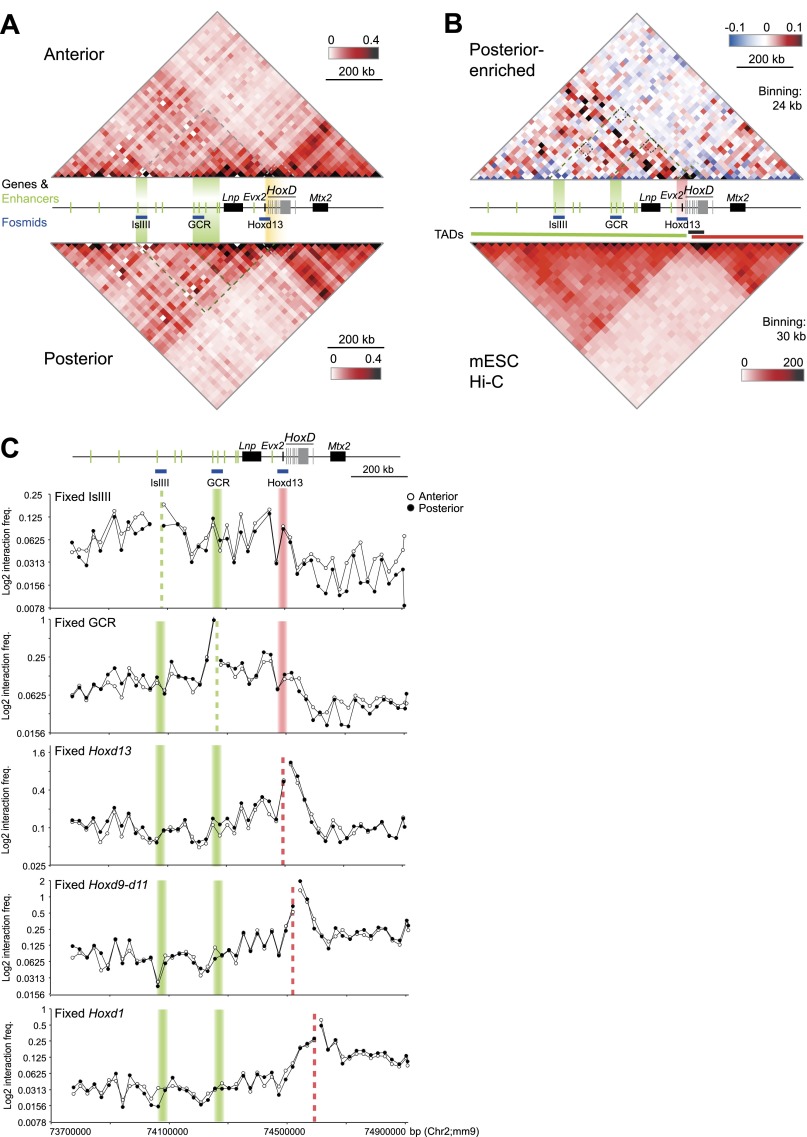
Long-range interactions in the 5′ *HoxD* regulatory region in anterior and posterior distal limbs. (*A*) 5C heat maps show the average interaction frequencies (24-kb bins) across *HoxD* and its regulatory domain in anterior (A2; *top*) and posterior (P2; *bottom*) limb cell lines according to color scales as described in Figure 1. Interaction frequencies were normalized based on total sequence read number. Black dotted lines indicate the areas encompassing IslIII–*Hoxd13* or GCR–*Hoxd13*. (*B*, *top*) Heat map showing 5C interactions enriched in P2 (red) or A2 (blue) cells. Heat map values are the difference of normalized interaction frequencies between P2 and A2 cells and are color-coded according to the scale at the *top right*. (*Bottom*) Heat map shows Hi-C data normalized based on read depth and the position of TADs (Dixon et al. 2012) for the corresponding region in mESCs. (*C*) Virtual 4C analysis obtained by extracting 5C interactions with viewpoints fixed at IslIII, GCR, *Hoxd13*, *Hoxd9–10*, and *Hoxd1*. Dashed lines indicate the position of the fixed viewpoint from regulatory elements (green) or *Hoxd* genes (orange). Data from A2 anterior and P2 posterior limb cell lines are in open and filled circles, respectively. Data from a biological replicate are shown in Supplemental Figure S4, and unprocessed normalized data are shown in Supplemental Figure S6B.

A key attribute of 4C is that ligation products are detected looking out from single viewpoints, and so the conformation of whole chromosomal domains cannot be extrapolated from these restricted data. We therefore used 5C to compare all of the cross-linked associations captured between sequences in the 1.2-Mb region containing *HoxD* and its 5′ regulatory domain (Fig. 3A) in A2 and P2 cells. Two interaction domains are clearly identified in both anterior and posterior limb cells (Fig. 4A; Supplemental Figs. S4A, S6B), demarcated by a boundary at *Hoxd9-10*. This corresponds well to the major TAD boundary reported in mESC Hi-C data (Fig. 4B, bottom heat map; Dixon et al. 2012). The 5′ *Hoxd* region, particularly around *Hoxd13*, has a high cross-linking efficiency with *Evx2* and *Lnp* and throughout the 5′ gene desert. In contrast, the 3′ end of *HoxD* predominantly interacts with the 3′ gene desert.

This aspect of *HoxD* compartmentalization is consistent with 4C results from E9.5 and E12.5 limbs (Montavon et al. 2011; Andrey et al. 2013). To examine this more closely, we extracted the interactome of IslIII, GCR, *Hoxd13*, *Hoxd9–11*, and *Hoxd1* from our 5C data sets and generated “virtual 4C” profiles by considering each of the fixed viewpoints as pseudobaits (Fig. 4C; Supplemental Fig. S4C). This is reminiscent of the approach used to compare interaction profiles derived from 4C and Hi-C in mESCs (Denholtz et al. 2013). Although virtual 4C confirms the generally higher *Hoxd9–10* and *Hoxd1* interaction frequencies with the region 3′ of *HoxD* compared with the 5′ flanking region, for the fixed viewpoint of *Hoxd13* interaction, frequencies up to 400 kb into the 5′ regulatory domain are not substantially higher than those extending 400 kb in the other direction. This argues against strictly exclusive conformational compartments for 5′ *Hoxd* and more 3′ regions in the limb and is not consistent with the 4C signals enriched throughout the region 5′ of *Hoxd13*, compared with the 3′ direction, during limb development (Andrey et al. 2013).

5C heat maps (Fig. 4AB; Supplemental Figs. S4AB) and virtual 4C (Fig. 4C; Supplemental Fig. S4C) also do not identify specific interactions between the regulatory elements of the 5′ gene desert and *Hoxd13* that would be indicative of discrete enhancer–promoter loops. Instead, the data point to chromatin conformations with enriched interaction frequencies between the enhancers (e.g., IslI, IslIII, and GCR) and the whole 5′ regulatory domain, including *Hoxd13* (Fig. 4A,B, larger area with dotted outline)*.* This is consistent with the view obtained by FISH of enhancers and target genes brought into proximity by a general compaction of the whole region rather than by specific loops (Fig. 3D; Williamson et al. 2012). For the region up to and just beyond the GCR, these interactions are generally more frequent in *Hoxd13*-expressing P2 cells than in A2 cells (Fig. 4B,C, smaller dotted area; Supplemental Fig. S4B,C). FISH also captures this difference (Fig. 3). In contrast, 5C interaction frequencies between IslIII and the rest of the regulatory domain extending toward *Hoxd13* are no different between the two cell lines (Fig. 4C), and this is also reflected in the FISH data.

### A compact HoxD domain in ESCs requires PRC2

Limb cells do not lend themselves readily to further dissection of the mechanisms that control chromatin structure. In contrast, in mESCs, the visibly compact chromatin structures of the silent *HoxB* and *HoxD* loci have been shown to be dependent on the PRC complexes PRC1 and PRC2 (Eskeland et al. 2010).

**Figure 5.**
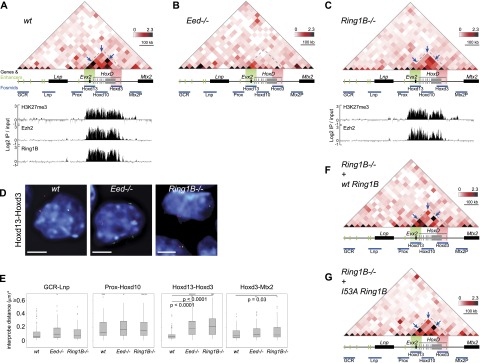
5C signals within the *HoxD* domain in polycomb mutant ESCs. 5C sequencing across *HoxD* in wild-type (wt) (*A*), PRC2 mutant (*Eed*^*−/−*^) (*B*), and PRC1 mutant (*Ring1B*^*−/−*^) (*C*) mESCs. 5C heat maps show the average interaction frequencies, normalized based on total sequence read number, per 20-kb bin using color scales as described in Figure 1. *Below* the heat maps, the position of genes is indicated in gray, regulatory elements are in green, and fosmid probes are in blue. Green and red highlight the regions covered by the fosmids used for the analysis in *D*. In *A* and *C*, ChIP–chip data for H3K27me3, Ezh2 (PRC2), and Ring1B (PRC1) from the respective mESCs are shown *below* the 5C heat maps (data from Illingworth et al. 2012). In *Eed*^*−/−*^ ESCs, Ezh2 is degraded, and H3K27me3 is reduced globally (Montgomery et al. 2005), so no ChIP data are shown. Data for a biological replicate are in Supplemental Figure S5. Unprocessed normalized data are shown in Supplemental Figure S7A. (*D*) 3D-FISH with Hoxd13 and Hoxd3 probe pairs (red and green) in nuclei (blue) from wild-type (wt) and polycomb-null mutant (*Eed*^*−/−*^ and *Ring1B*^*−/−*^) mESCs. Bars, 5 μm. (*E*) Box plots showing the distribution of 3D-FISH squared interprobe distances (*d*^2^) for probe pairs GCR–Lnp, Prox–Hoxd10, Hoxd13–Hoxd3, and Hoxd3–Mtx2P in wild-type and polycomb-null mutant (*Eed*^*−/−*^ and *Ring1B*^*−/−*^) ESCs. Boxes show the median and interquartile range of the data; crosses signify outliers. *n* = 93–107 loci. The statistical significances between the probe pairs covering the same region in different cells were examined by Mann-Whitney *U*-tests. (*F*,*G*) 5C sequencing across *HoxD* in *Ring1B*^*−/−*^ mESCs rescued with wild-type Ring1B (*F*) and I53A mutant Ring1B (*G*) (Eskeland et al. 2010). 5C heat maps show the mean interaction frequencies per 20-kb bin. Data for a biological replicate are in Supplemental Figure S5. Unprocessed normalized data are shown in Supplemental Figure S7B.

H3K27me3 is catalyzed by the PRC2 component Ezh2, whose chromatin immunoprecipitation (ChIP) profile across *HoxD* in mESCs also parallels that of H3K27me3 (Fig. 5A; Illingworth et al. 2012). The distribution of H3K27me3 and Ezh2 also corresponds to the extent of the strong 5C interaction domain, suggesting that PRC2 might be important for chromatin conformation at *HoxD* (Fig. 5A; Supplemental Figs. S5A, S7). To directly demonstrate a role of PRC2 in chromatin compaction, we carried out both 5C and 3D-FISH on ESCs mutant for PRC2 (*Eed*^*−/−*^). Ezh2 is degraded (Montgomery et al. 2005), and H3K27me3 is lost globally from these cells (Eskeland et al. 2010). Ring1B (PRC1) binding is also lost from *Hox* loci in PRC2-null cells (Eskeland et al. 2010; Leeb et al. 2010), likely due to the dependence on H3K27me3 recognition by the Cbx subunits of canonical PRC1 (Morey et al. 2012). The 5C interaction domain over *HoxD* was virtually eliminated in *Eed*^*−/−*^ cells (Fig. 5B; Supplemental Fig. S5B), coincident with a visible decompaction that is detected by FISH and restricted to *HoxD* (Fig. 5D,E, *Hoxd13*–*Hoxd3*; Supplemental Table S7, wt/*EED*^*−/−*^, *P* < 0.0001; Eskeland et al. 2010). Therefore, both FISH and 5C appear to show that PRC2 is required to maintain *Hox* loci in a compact silent chromatin conformation in mESCs.

### 5C and FISH data are discordant in PRC1 mutant ESCs

**Figure 6.**
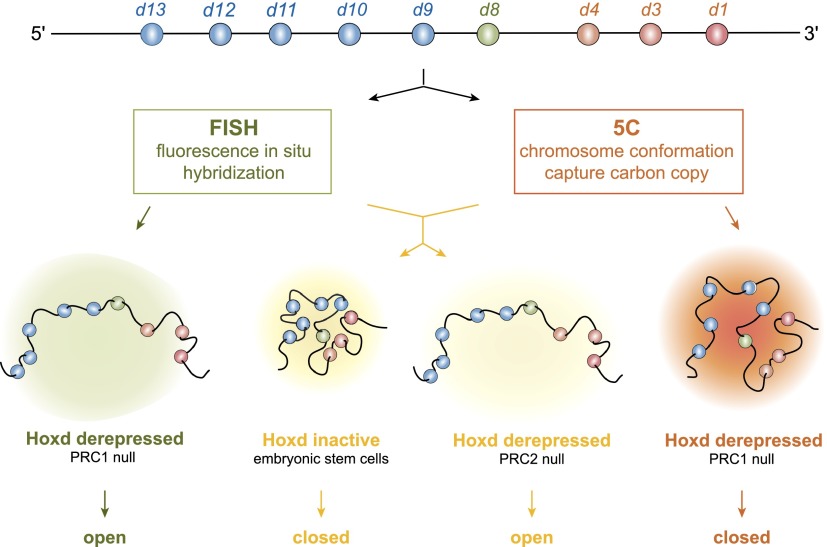
FISH and 5C analysis can yield compatible or discordant chromatin topographies at high resolution. (*Top*) Schematic of the *HoxD* locus showing the 5′ (blue) and 3′ (orange) *Hoxd* genes. (*Bottom*) The views of chromatin topography for *HoxD* extrapolated from FISH or 5C data are shown for wild-type and polycomb mutant ESCs. (*Middle*) For wild-type and PRC2-null cells, FISH and 5C give coherent views of a compact (wild-type) versus unfolded (PRC2-null) chromatin conformation. However, in the case of PRC1-null cells, FISH (*left*) indicates an unfolded chromatin conformation similar to that seen for PRC2-null cells, whereas 5C (*right*) suggests a much more tightly folded domain.

Binding of the Ring1B PRC1 subunit across *HoxD* parallels that of both H3K27me3 and Ezh2 in wild-type mESCs (Fig. 5A; Illingworth et al. 2012). PRC2 is necessary but not sufficient for chromatin compaction at *HoxD*. In *Ring1B*^*−/−*^ mESCs, *HoxD* visibly decompacts relative to the wild type (*P* < 0.0001) to the same extent as that seen in the absence of PRC2; the interquartile range of interprobe distances in *Ring1B*^*−/−*^ cells (300–569 nm) is similar to that in *Eed*^*−/−*^ cells (300–539 nm), and both are different from wild-type mESCs (192–300 nm) (Fig. 5D,E; Supplemental Table S7). *Hox* genes are also activated despite the persistence of H3K27me3 and PRC2 at these loci (Fig. 5C; Eskeland et al. 2010; Illingworth et al. 2012). Thus, we expected that 5C data from *Ring1B*^*−/−*^ mESCs would resemble those from PRC2 mutant cells. Instead, high 5C contacts frequencies remain across *HoxD* in *Ring1B*^*−/−*^ mESCs in a pattern similar to that seen in wild-type cells (Fig. 5C, arrow; Supplemental Figs. S5C, S7). Thus, if interaction frequency is considered as inversely proportional to spatial distance, the 5C data would suggest that *HoxD* remains at least partially folded in the absence of Ring1B, which is in conflict with the decompact *HoxD* domain seen by FISH (Figs. 5D,E, 6). Moreover, in *Ring1B*^*−/−*^ cells, 5C data appear to indicate that the *HoxD* locus itself is the most highly folded part of the entire region under investigation (Fig. 5C), whereas, to the contrary, FISH suggests that *HoxD* is the most unfolded part of the whole region in these mutant cells (Fig. 5E).

We previously showed that Ring1B reintroduced into *Ring1B*^*−/−*^ cells recapitulates the binding profile of the endogenous protein across *HoxD* and restores chromatin compaction as measured by FISH (Eskeland et al. 2010). Strong and reproducible 5C contacts are also restored to a pattern comparable with wild-type cells at *HoxD* in the cells rescued with wild-type *Ring1B* (Fig. 5F, arrow; Supplemental Fig. S5D,S7). Chromatin folding and compaction at *HoxD* does not require the enzymatic activity of Ring1B, since complementation of *Ring1B*^*−/−*^ cells with the ubiquitination-deficient *Ring1B* I53A mutant also restores both the levels and the pattern of 5C contacts (Fig. 5G, arrow). These results are consistent with our previous study demonstrating that Ring1B visibly compacts chromatin structure at *Hox* loci in ESCs independent of its histone ubiquitination activity (Eskeland et al. 2010).

## Discussion

With rather few exceptions (Kalhor et al. 2011; Nora et al. 2012; Giorgetti et al. 2014), there have been limited systematic and detailed comparisons between 3C-type data sets and cytological assays of 3D chromatin organization. Here we directly compared 5C and FISH data across a region of the mouse genome encompassing the *HoxD* locus in different cell types and activity states and in the absence of major epigenetic regulators of the locus. We identified some situations in which the 5C and FISH data are in agreement, as, for example, in undifferentiated mESCs (Figs. 1, 2; Supplemental Fig. S1) and in the absence of the PRC2 complex (Fig. 5; Supplemental Figs. S5, S7). However, in other cases, such as upon gene activation during ESC differentiation (Fig. 2; Supplemental Fig. S1) or mutation of the PRC1 complex (Fig. 5; Supplemental Fig. S5), domains of elevated 5C cross-linking frequencies conflict with the visible decompaction of these same regions observed by FISH (Fig. 6). This suggests that, at high resolution, 3C interaction frequency might not always simply reflect physical distances. This is important to consider if 3C-type information is ever to be used in modeling genome organization at the level of sub-TADs (Nora et al. 2013) and beyond.

### 5C interactions of active Hox genes and nuclear organization

In undifferentiated mESCs, 5C data identify a domain of robust interactions across *HoxD* that is consistent with its visibly compact structure relative to the surrounding gene deserts (Fig. 1B; Supplemental Fig. S1A; Morey et al. 2007). Upon RA-induced differentiation and activation of 3′ *Hoxd* genes, 5C interactions within *HoxD* are reduced compared with undifferentiated mESCs (Fig. 2; Supplemental Figs. S1, S6). This is consistent with FISH analysis showing chromatin unfolding strictly at *HoxD* and not at the flanking regions (Fig. 2). However, the 3′ *Hoxd* region simultaneously gains 5C contacts with the upstream gene desert region, while there are no significant reductions in distances detectable by FISH, which might have been expected if enhanced cross-linking simply arose from spatial proximity (Fig. 2D).

What does visibly change upon mESC differentiation is the nuclear localization of both the 3′ *Hoxd* genes and the upstream gene desert as they gain the ability to loop out of the territory occupied by the bulk of chromosome 2 (Morey et al. 2007, 2009). Looping out from CTs is dynamic (Müller et al. 2010), and, genome-wide, the ability of sequences to become cross-linked and captured to distant sequences—even those located on other chromosomes—by Hi-C corresponds remarkably well to the looping-out frequency (Kalhor et al. 2011). We suggest that access to this nuclear compartment outside of the CT core rather than actual physical distance per se may be responsible for the high 5C contact frequencies between 3′ *Hoxd* genes and the upstream genomic region in differentiated mESCs. The fact that 3C ligation products originate mainly from insoluble aggregates within unlysed swollen nuclei rather than on soluble chromatin (Gavrilov et al. 2013c) raises the possibility that nuclear structures enhance or promote 3C associations, as was suggested for the active *β-globin* gene and its enhancer (Gavrilov et al. 2013a). This may also explain why higher 3C contact frequencies and inferred structurally compact domains were previously found associated with activated *Hox* loci in human fibroblasts (Wang et al. 2011) and mouse embryos (Noordermeer et al. 2011, 2014).

### High 5C frequencies within a visibly decondensed chromatin region

The compact structure of *Hox* loci in mESCs depends on the PRCs (Eskeland et al. 2010), and the *HoxD* 5C interaction domain in mESCs (Fig. 1) almost exactly corresponds to that of Ezh2 (PRC2) binding and H3K27me3 enrichment (Fig. 5A; Illingworth et al. 2012). We demonstrated the role of PRC2 in maintaining this compact domain by showing that, in PRC2 *Eed*^*−/−*^ mutant mESCs, 5C domain interactions are completely lost and that chromatin unfolding visualized by FISH is restricted to the *HoxD* cluster itself (Fig. 5B; Supplemental Fig. S5B, S7). To our knowledge, this is the first instance in which the genetic deletion of a major epigenetic regulator has been shown to have such a profound effect of almost eliminating a 3C interaction domain, and, in this instance, both 5C and FISH results are concordant (Fig. 6). However, this is not the case for the data from PRC1 *Ring1B*^*−/−*^ mutant mESCs, where FISH indicates an unfolding of *HoxD* to an extent similar to that seen in PRC2-null cells, yet a 5C interaction domain—albeit weakened compared with the wild type—remains over *HoxD* (Fig. 5C; Supplemental Fig. S5C). These data suggest that 5C can capture substantial cross-linked ligation products from within a visibly unfolded chromatin domain.

Chromatin composition might provide one explanation for this. While *HoxD* remains coated with PRC2 proteins in *Ring1B*^*−/−*^ cells (Illingworth et al. 2012), it is completely devoid of them in *Eed*^*−/−*^ cells (Eskeland et al. 2010; Leeb et al. 2010). Formaldehyde preferentially forms cross-links with lysine, tryptophan, and cysteine side chains in proteins (Toews et al. 2008). Just considering the core components of PRC2 (Eed, EzH2, and Suz12), each molecule of the complex provides >200 extra of these reactive residues, including >150 lysine residues alone. While the stoichiometry of PRC2 per nucleosome is not known, given the blanket ChIP signal for PRC2 across *HoxD*, it is plausible that the thousands of extra formaldehyde cross-linkable amino acid side chains present in PRC1 mutant cells due to the persistent binding of PRC2 are responsible for the elevated 5C signals in a structure that is much more open than the data would suggest. Similarly, the residual levels of H3K27me3 at the 5′ end of *HoxD* could also explain why, despite FISH indicating that *HoxD* is the most decondensed part of the region under study in differentiated ESCs, 5C signals across *HoxD* generally exceed those outside of this region (Fig. 2). Another confounding factor could be the low frequency of ligation products produced in 3C reactions, even for enhancer–promoter interactions, relative to the total number of fragments present (Gavrilov et al. 2013b). If this represents the bona fide frequency of interaction between two genomic loci, then it may be below the level at which FISH is able to distinguish this from background. While we raised these issues from our study of *HoxD* using 5C and FISH, there may be different factors that could influence the interpretation of FISH with other 3C data (4C or Hi-C) and the outcome of these studies at other genomic loci with different compaction states and chromatin flavors.

We conclude that, at high resolution, products captured by 3C do not always simply represent spatial proximity or molecular interaction between two DNA sequences but may arise from indirect cross-linking of chromatin fragments that may be hundreds of nanometers apart in nuclear space in the original cells, perhaps exacerbated by the partial chromatin decondensation that accompanies the initial digestion and SDS steps of 3C prior to ligation (Gavrilov et al. 2013c). This can be compounded by differences in the nuclear environment of the regions and/or the protein composition of the chromatin fiber. This is unlikely to be an issue when only considering low-resolution views of spatial nuclear organization. Of course, we cannot exclude the possibility that the FISH technique is also contributing to the apparent conflict in the data at high resolution. This could be due to an inability of FISH to capture weak or transient interactions or to perturbations to chromatin ultrastructure as a result of the heat denaturation step. Nonetheless, we think that FISH quite faithfully reflects the chromatin compaction state because, in all cases in which a direct comparison has been made, the data inferred from FISH are completely consistent with analysis of the same regions in vivo in living cells (Müller et al. 2010) and with the biophysical properties of chromatin from those same regions assayed in vitro (Gilbert et al. 2004; Naughton et al. 2010). Moreover, a careful quantitative analysis by superresolution microscopy before and after 3D-FISH shows evidence that FISH preserves many aspects of chromatin structure and organization (Markaki et al. 2012).

A decade ago, it was realized that the formation of cross-linked nuclear aggregates or “nuclear crumbs” presented problems in extrapolating data from formaldehyde cross-linking to nuclear organization (Schmid et al. 2004; Belmont 2014), and our data support that view. We suggest that visual and molecular approaches are complementary to each other and that models of 3D genome organization should be extrapolated from data validated by independent methods.

## Materials and methods

### Cell culture

Wild-type (WTF18), *Ring1B*^−/−^ (Leeb and Wutz 2007), and *Eed* mutant (17Rn5-3354SB; *Eed*^−/−^ B1.3 and *Eed*^−/−^ G8.1) (Azuara et al. 2006) mESCs were grown on mitomycin C-treated primary embryonic fibroblasts (PEFs) derived from E12.5 mouse embryos as described (Eskeland et al. 2010). OS25 ESCs were cultured and differentiated for 3 d (2 d with RA) as described (Morey et al. 2007). For FISH and 5C analysis, ESCs were trypsinized, and the PEFs were removed by allowing them to reattach to the tissue culture plastic twice for 30 min in LIF-containing medium.

Mesenchymal cell lines derived from Immortomouse E10.5 anterior (A2) and posterior (P2) distal forelimb buds were cultured as described previously (Williamson et al. 2012).

### FISH

Paraformaldehyde (pFA)-fixed cells were permeabilized in 0.5% Triton X-100, washed in PBS, and stored at −80°C. 3D-FISH was carried out as previously described (Eskeland et al. 2010). Fosmid clones used as FISH probes are listed in Supplemental Table S1.

Slides were imaged and analyzed as described previously (Williamson et al. 2012). The statistical significance of differences in interprobe distances was assessed using the nonparametric Mann-Whitney *U*-test. Each data set consisted of between ∼80 and 120 loci for each cell line and each probe combination.

### 3C library preparation

Cells were fixed with 1% formaldehyde for 10 min at room temperature. Cross-linking was stopped with 125 mM glycine for 5 min at room temperature followed by 15 min on ice. Cells were centrifuged at 400*g* for 10 min at 4°C, supernatants were removed, and cell pellets were flash-frozen on dry ice.

Cell pellets were treated as previously described (Dostie and Dekker 2007; Ferraiuolo et al. 2010). Briefly, 10 million to 20 million fixed cells were incubated for 15 min on ice in 200 μL of lysis buffer (10 mM Tris at pH 8.0, 10 mM NaCl, 0.2% NP40, supplemented with fresh protease inhibitor cocktail). Cells were then disrupted on ice with a dounce homogenizer (pestle B; 2 × 20 strokes); cell suspensions were transferred to Eppendorf tubes and centrifuged at 2000*g* for 5 min. Supernatants were removed, the cell pellets were washed twice with 100 μL of 1× EcoRI buffer (New England Biolabs), and the cell pellet was resuspended in 100 μL of 1× EcoRI buffer and divided into two Eppendorf tubes. We added 1× EcoRI buffer (337 μL) to each tube, and the mixture was incubated for 10 min at 65°C with 0.1% SDS. Forty-four microliters of 10% Triton X-100 was added before overnight digestion with 400 U of EcoRI. The restriction enzyme was then inactivated by adding 86 μL of 10% SDS and incubation for 30 min at 65°C. Samples were then individually diluted into 7.62 mL of ligation mix (750 μL of 10% Triton X-100, 750 μL of 10× ligation buffer, 80 μL of 10 mg/mL of BSA, 80 μL of 100 mM ATP, 3000 cohesive end units of T4 DNA ligase) and incubated at for 2 h 16°C.

3C libraries were incubated overnight at 65°C with 50 μL of Proteinase K (10 mg/mL) and an additional 50 μL of Proteinase K the following day for 2 h. The DNA was purified by one phenol and one phenol–chloroform extraction and precipitated with 0.1 vol (800 μL) of 3 M NaOAc (pH 5.2) and 2.5 vol of cold EtOH (20 mL). After at least 1 h at −80°C, the DNA was centrifuged at 20,000*g* for 25 min at 4°C, and the pellets were washed with cold 70% EtOH. DNA was resuspended in 400 μL of TE (pH 8.0) and transferred to Eppendorf tubes for another phenol–chloroform extraction and precipitation with 40 μL of 3 M NaOAc (pH 5.2) and 1.1 mL of cold EtOH. DNA was recovered by centrifugation and washed eight times with cold 70% EtOH. Pellets were then dissolved in 100 μL of TE (pH 8.0) and incubated with1 μL of 10 mg/mL RNase A for 15 min at 37°C.

### 5C primer and library design

5C primers covering the *USP22* (mm9, chr11: 60,917,307–61,017,307) and *HoxD* (mm9, chr2: 73,750,000–74,910,000) regions were designed using my5C.primer (Lajoie et al. 2009) and the following parameters: optimal primer length of 30 nucleotides (nt), optimal TM of 65°C, and default primer quality parameters (mer: 800, U-blast: 3, S-blasr: 50). Primers were not designed for large (>20-kb) and small (<100-bp) restriction fragments, low-complexity and repetitive sequences, or when there were sequence matches to more than one genomic target. The *USP22* regions was used to assess the success of each 5C experiment but was not used for further data normalization or quantification.

The universal A-key (CCATCTCATCCCTGCGTGTCTCCGACTCAG-[5C-specific]) and the P1-key tails ([5C-specific]-ATCACCGACTGCCCATAGAGAGG) were added to the forward and reverse 5C primers, respectively. Reverse 5C primers were phosphorylated at their 5′ ends. An alternating design consisting of 133 primers in the *HoxD* region (66 forward and 67 reverse primers) was used for analysis of ESCs. An extended alternating design of 203 primers (99 forward and 104 reverse primers) was used for the limb cells. Primer sequences are listed in Supplemental Table S8.

### 5C library preparation

5C libraries were prepared and amplified with the A-key and P1-key primers as described previously (Fraser et al. 2012). Briefly, 3C libraries were first titrated by PCR for quality control (single band, absence of primer dimers, etc.) and to verify that contacts were amplified at frequencies similar to that usually obtained from comparable libraries (same DNA amount from the same species and karyotype) (Dostie and Dekker 2007; Dostie et al. 2007, Fraser et al. 2010). In general, we used 1–11 μg of 3C library per 5C ligation reaction.

5C primer stocks (20 μM) were diluted individually in water on ice and mixed to a final concentration of 0.002 μM. Mixed diluted primers (1.7 μL) were combined with 1 μL of annealing buffer (10× NEBuffer 4 [New England Biolabs]) on ice in reaction tubes. Salmon testis DNA (1.5 μg) was added to each tube, followed by the 3C libraries and water to a final volume of 10 μL. Samples were denatured for 5 min at 95°C and annealed for 16 h at 48°C. Ligation with 10 U of Taq DNA ligase was performed for 1 h at 48°C. One-tenth (3 μL) of each ligation was then PCR-amplified individually with primers against the A-key and P1-key primer tails. We used 26 or 28 cycles based on dilution series showing linear PCR amplification within that cycle range. The products from two to four PCR reactions were pooled before purifying the DNA on MinElute columns (Qiagen).

5C libraries were quantified on agarose gels and diluted to 0.0534 ng/μL (for Xpress template kit version 2.0) or 0.0216 ng/μL (for ion PGM template OT2 200 kit). One microliter of diluted 5C library was used for sequencing with an ion PGM sequencer. Samples were sequenced onto ion 316 chips following either the ion Xpress template kit version 2.0 and ion sequencing kit version 2.0 protocols or the ion PGM template OT2 200 kit and ion PGM sequencing 200 kit version 2.0 protocols as recommended by the manufacturer (Life Technologies).

### 5C data analysis

Analysis of the 5C sequencing data was performed as described earlier (Berlivet et al. 2013). The sequencing data were processed through a Torrent 5C data transformation pipeline on Galaxy (https://main.g2.bx.psu.edu). Posterior-enriched and D3-enriched 5C interactions were obtained by subtracting anterior and undifferentiated 5C sequencing data, respectively. Data were normalized by dividing the number of reads of each 5C contact by the total number of reads from the corresponding sequence run. All scales correspond to this ratio multiplied by 10^3^. The numbers of total reads and used reads are provided for each experiment in Supplemental Table S9. The unprocessed heat maps of the normalized 5C data sets can be found in Supplemental Figures S6 and S7. 5C data sets (accession no. GSE61814) can be downloaded from the Gene Expression Omnibus (GEO) (http://www.ncbi.nlm.nih.gov/geo).

## Supplementary Material

Supplemental Material
