# Supplementary material for: Spatial genome organization: contrasting views from chromosome conformation capture and fluorescence in situ hybridization
Source: Genes Dev. 2014 Dec 15;28(24):2778–91. doi: 10.1101/gad.251694.114 (PMC4265680; doi:10.1101/gad.251694.114)
Supplement: Supplemental Material [file supp_28_24_2778__index.html]

Supplemental Material 

# Spatial genome organization: contrasting views from chromosome conformation capture and fluorescence in situ hybridization

## Supplemental Material

**Files in this Data Supplement:**

- Supplemental Figures.pdf
