# Supplementary figures and images for: Spatial genome organization: contrasting views from chromosome conformation capture and fluorescence in situ hybridization
Source: Genes Dev. 2014 Dec 15;28(24):2778–91. doi: 10.1101/gad.251694.114 (PMC4265680; doi:10.1101/gad.251694.114)

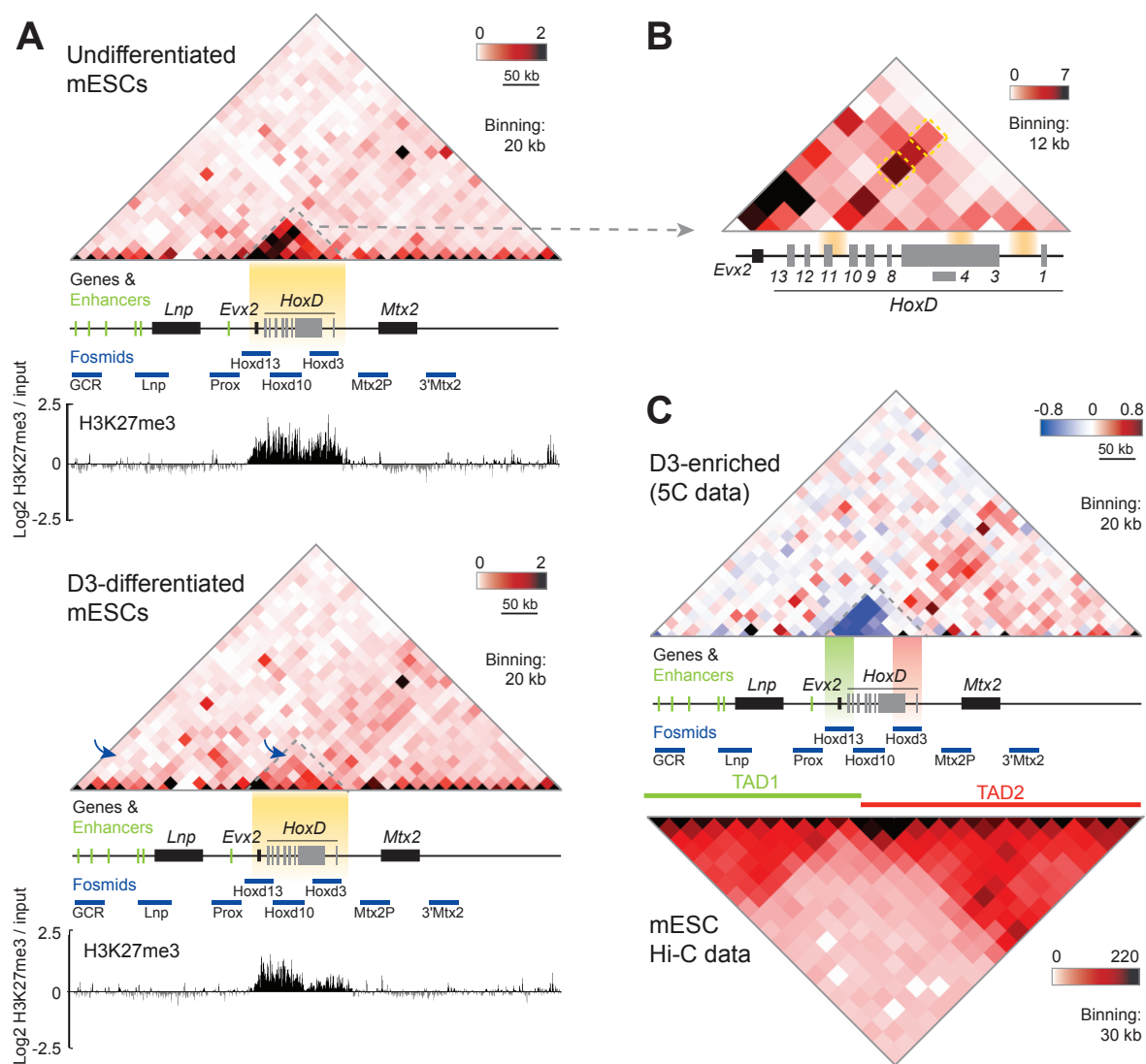

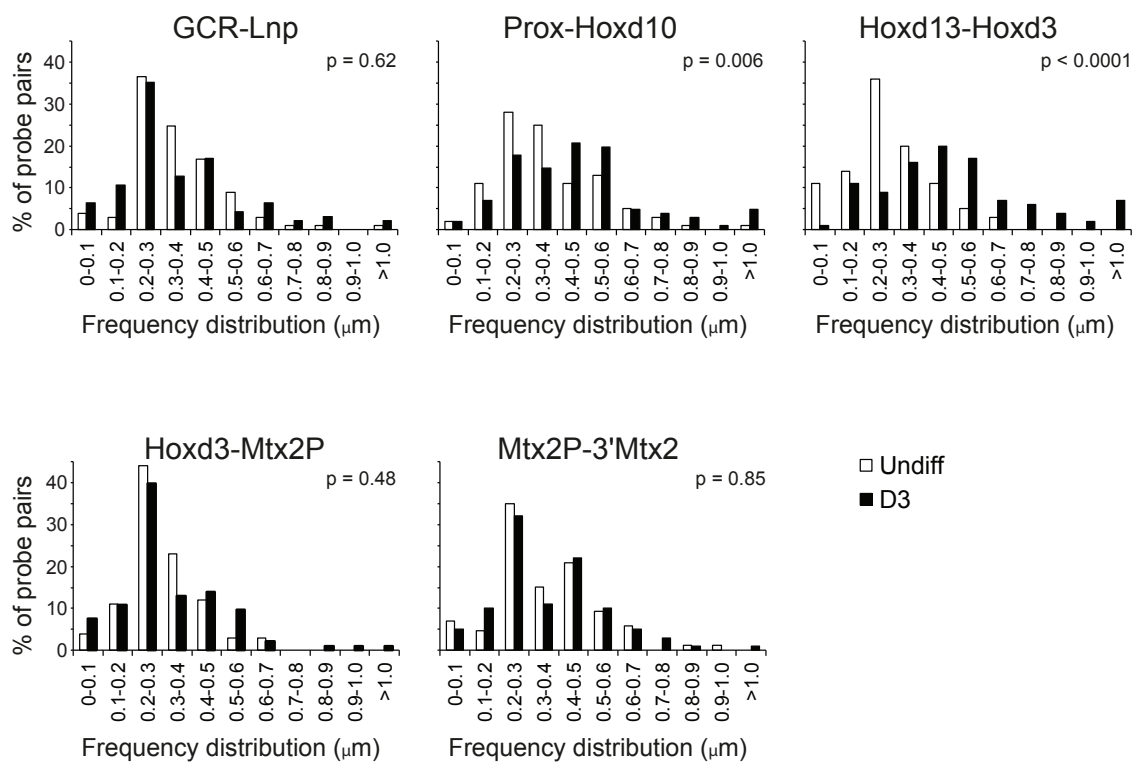

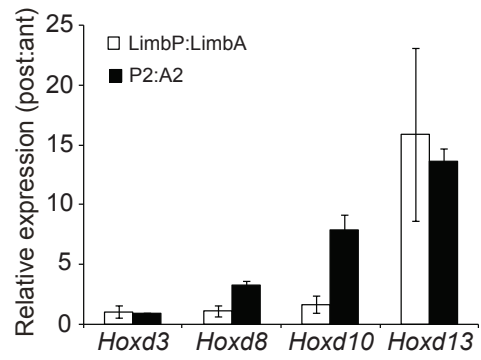

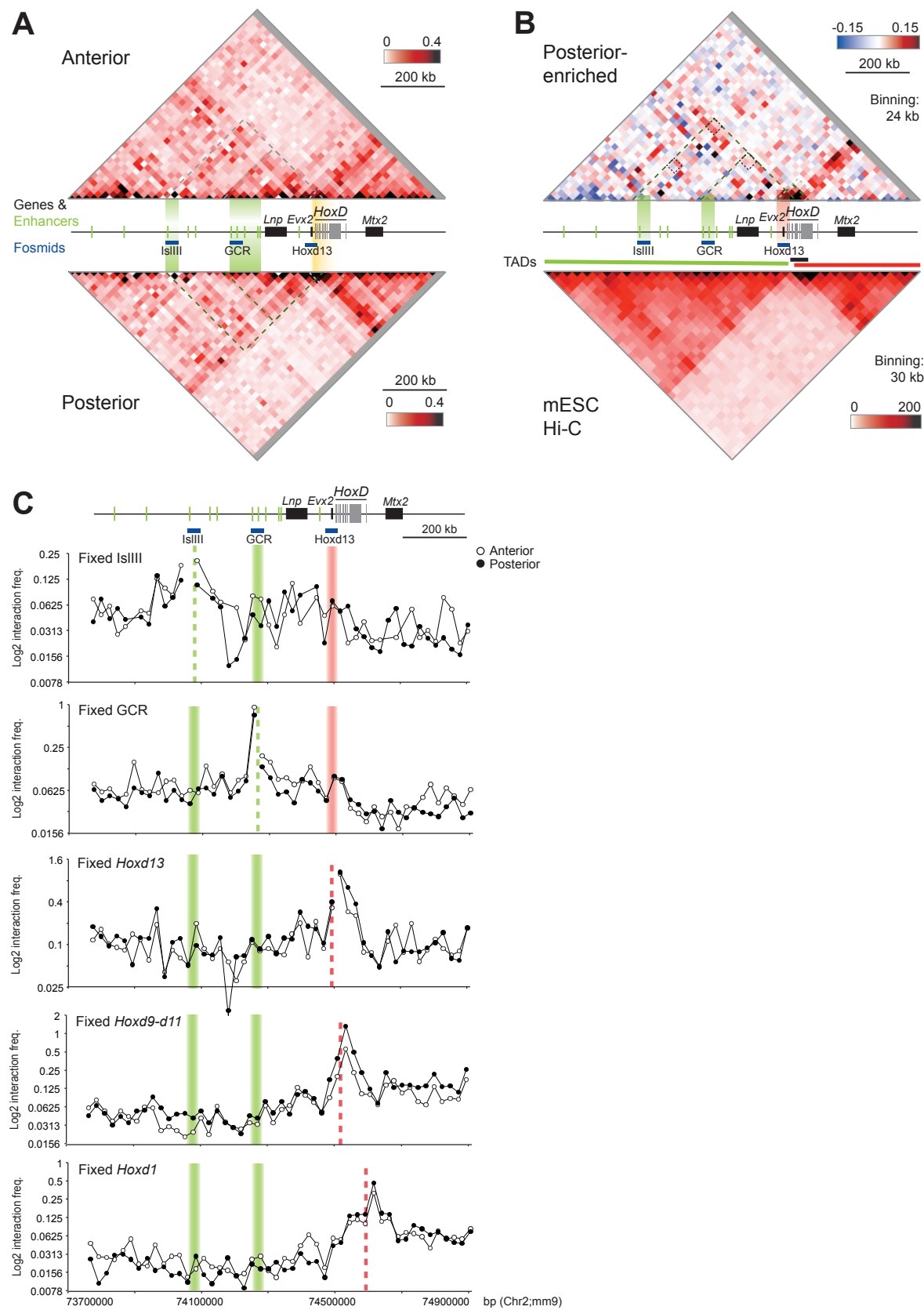

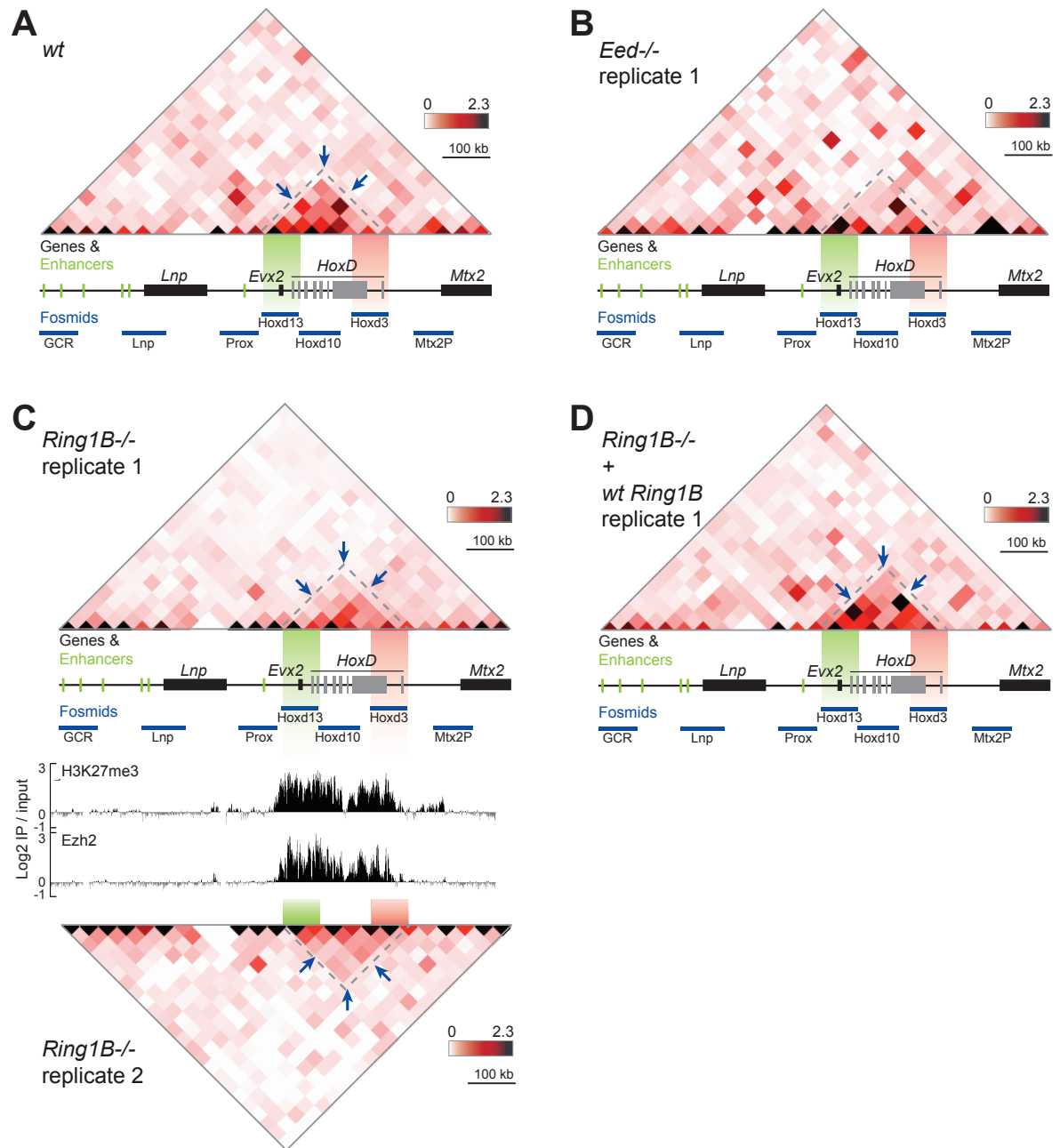

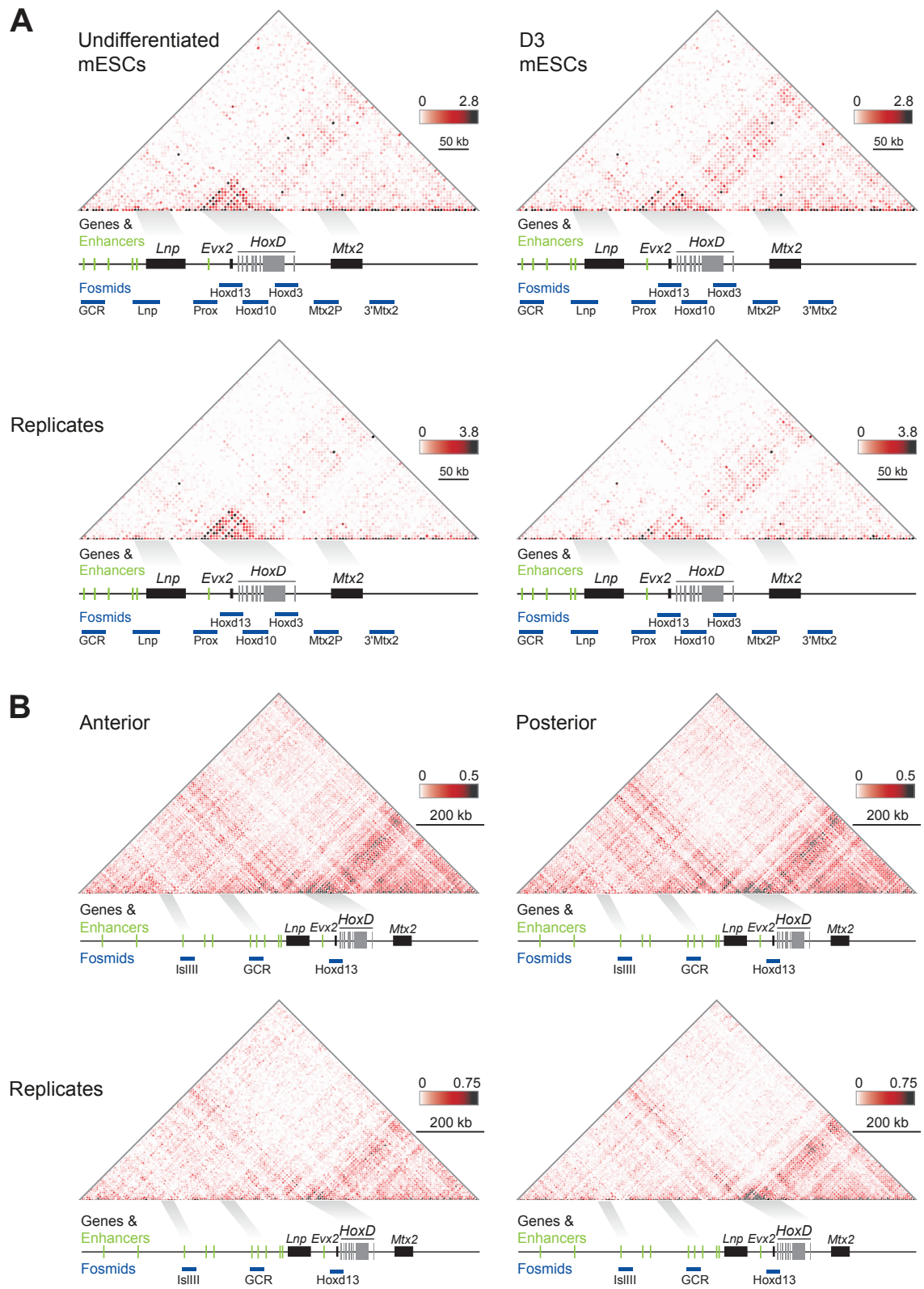

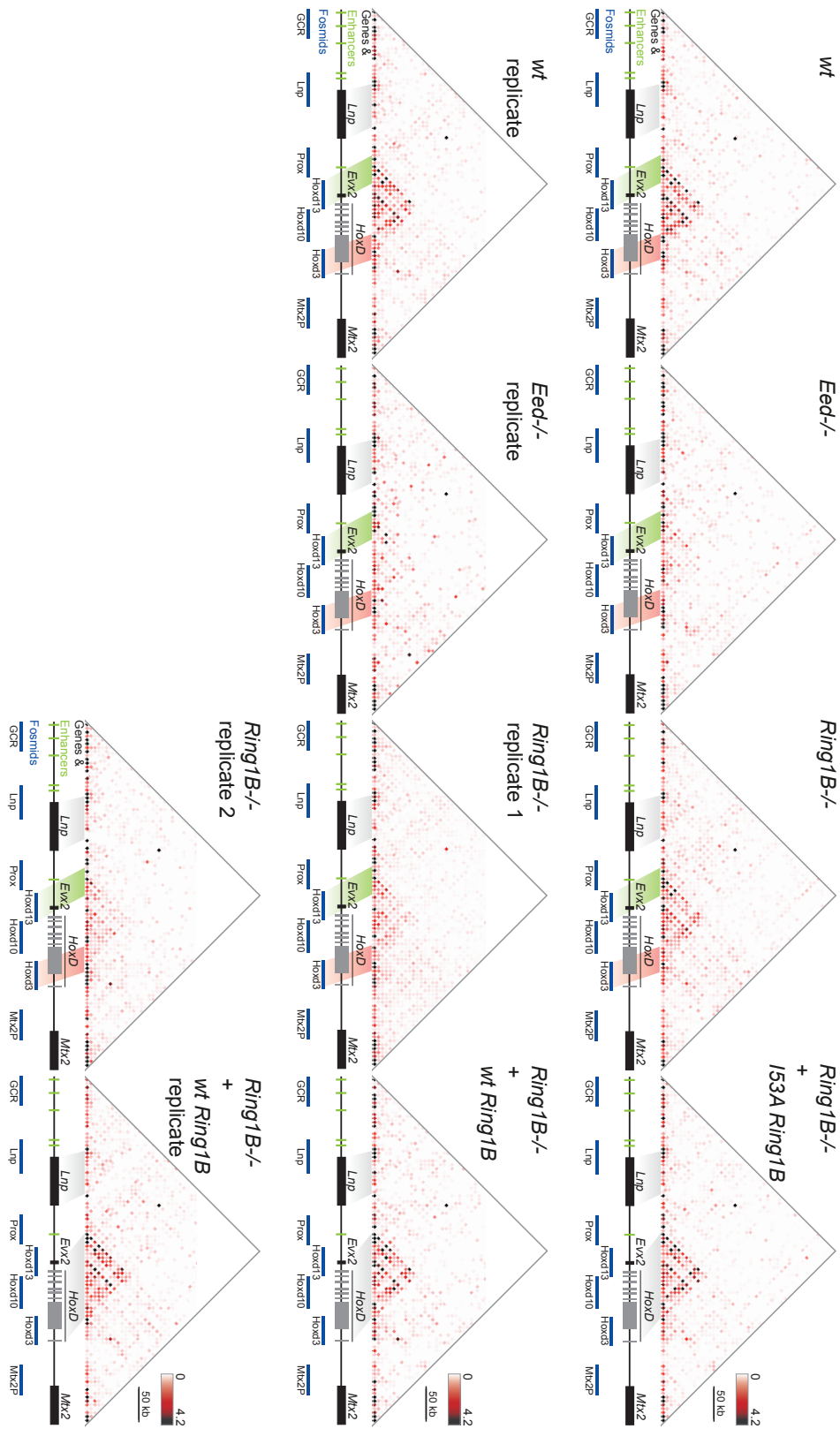

Supplement: Supplemental Material [file supp_28.24.2778_Supplemental_Figures.pdf]
